# An Examination of Dynamic Gene Expression Changes in the Mouse Brain During Pregnancy and the Postpartum Period

**DOI:** 10.1534/g3.115.020982

**Published:** 2015-11-20

**Authors:** Surjyendu Ray, Ruei-Ying Tzeng, Lisa M. DiCarlo, Joseph L. Bundy, Cynthia Vied, Gary Tyson, Richard Nowakowski, Michelle N. Arbeitman

**Affiliations:** * Department of Biomedical Sciences, Florida State University, College of Medicine, Tallahassee, Florida 32306; † Center for Genomics and Personalized Medicine, Florida State University, Tallahassee, Florida 32306; ‡ Department of Computer Science, Florida State University, Tallahassee, Florida 32306

**Keywords:** pregnancy, parturition, postpartum, RNA-seq, mouse, gene expression, Genetics of Sex

## Abstract

The developmental transition to motherhood requires gene expression changes that alter the brain to drive the female to perform maternal behaviors. We broadly examined the global transcriptional response in the mouse maternal brain, by examining four brain regions: hypothalamus, hippocampus, neocortex, and cerebellum, in virgin females, two pregnancy time points, and three postpartum time points. We find that overall there are hundreds of differentially expressed genes, but each brain region and time point shows a unique molecular signature, with only 49 genes differentially expressed in all four regions. Interestingly, a set of “early-response genes” is repressed in all brain regions during pregnancy and postpartum stages. Several genes previously implicated in underlying postpartum depression change expression. This study serves as an atlas of gene expression changes in the maternal brain, with the results demonstrating that pregnancy, parturition, and postpartum maternal experience substantially impact diverse brain regions.

The developmental transformation of the mammalian female brain during pregnancy, parturition, and the postpartum period prepare and drive maternal behaviors, and is both a response to, and presumably a cause of, the physiological changes accompanying these reproductive transitions (reviewed in Brunton and Russell 2008; Dulac *et al.* 2014; Hillerer *et al.* 2014; Kinsley and Amory-Meyer 2011). While it is clear that fluctuating titers of hormones contribute to changes in gene expression that mediate this transformation, how the female brain is shaped as a result of reproductive and maternal experience is not understood at a molecular-genetic or neural circuit level.

Hormones are vital for both the establishment and maintenance of pregnancy, parturition, and the onset of postpartum maternal behavior, with peptide hormones prolactin (Prl) and oxytocin (Oxt), and steroid hormones estrogen (E) and progesterone (P4) having key roles (reviewed in Brunton and Russell 2010). With respect to the brain, the epicenter of hormone regulation is the hypothalamus, which is the major central nervous system (CNS) component of the hypothalamus-pituitary-gonadal axis (reviewed in Brunton and Russell 2008, 2010; Russell *et al.* 2001). Hormones produced in the hypothalamus regulate the pituitary gland, which in turn regulates gonadal hormone production. The hormone feedback loops mean that the cells of the hypothalamus are both the source and the target of a variety of hormones.

Both positive and negative feedback loops are known to regulate the key hormones of pregnancy. The hormones luteinizing hormone (LH), follicle stimulating hormone (FSH) and Prl are released from the pituitary, E and P4 from the ovary, and Oxt, which regulates both labor and lactation, is produced in the hypothalamus. The central connections of the hypothalamus to other brain regions means that it also plays a fundamental role in regulating maternal and other behaviors. For example, the neural circuits that underlie maternal behaviors are primed by E and P4 during pregnancy and postpartum (Bridges 1984; Bridges *et al.* 1990; Gonzalez-Mariscal *et al.* 1996). Even the most basic mammalian maternal behavior, such as nursing, requires Prl to stimulate milk secretion (Horseman *et al.* 1997), and Oxt to stimulate milk ejection in response to suckling (reviewed in Brunton and Russell 2010).

Outside of the hypothalamus, and the hypothalamus-pituitary-gonadal axis, many brain areas are the presumed targets of hormones and play a role in maternal behavior. For example, lesions of the cingulate cortex, amygdala, septal regions, and regions of the brain that process olfactory information have been shown to impact maternal behaviors, such as pup retrieval and nest building (Kinsley and Amory-Meyer 2011; Slotnick 1967; Slotnick and Nigrosh 1975). These major reproductive transitions also impact cognition. For example, the hormone changes during pregnancy and the postpartum period are thought to underlie improved cognitive abilities, with reproductive experience enhancing spatial memory (reviewed in Kinsley *et al.* 1999), as well as other hippocampal functions (reviewed in Galea *et al.* 2014).

Previous gene expression studies that examined expression changes associated with the maternal brain have focused on single time points and examined particular subregions of the brain (Eisinger *et al.* 2013; Zhao *et al.* 2012), or particular gene functional groups, like neurotransmitter receptor pathway genes (Mann 2014). Thus far, there has not been a comprehensive examination of the brain to understand how extensive the molecular changes are in different brain regions, and also how different these changes are at each stage.

To understand how extensive the gene expression changes are, we have examined gene expression in four brain regions, using RNA-sequencing (RNA-seq). The rationale for the four brain regions examined are: (1) the hypothalamus: because of its key role in regulating hormones, and because it is both a source and target of many hormones; (2) the hippocampus; and (3) the neocortex: because of their roles in memory, learning, and cognition; and (4) the cerebellum: a key motor control region that, although it is known to have cells with hormone receptors, does not seem *a priori* to have a major role in the regulation of maternal behaviors. Thus, these four brain regions include regions previously implicated in underlying maternal behaviors, and those with less established roles. In this study, we examined these brain regions in virgin, pregnant, and postpartum females to provide a sequential view of gene expression changes across a changing physiological landscape, with a focus on the peripartum period, pregnancy time points near parturition, and early postpartum time points when the potential for maternal behaviors are likely being established, and also a later postpartum time point when maternal behaviors are robustly displayed.

## Materials and Methods

### Animal Husbandry

Eight-week-old male and female C57BL/6J (B6) mice were obtained from the Jackson Laboratory (Bar Harbor, ME). To generate timed pregnant mice, nulliparous B6 females were trio bred to B6 males. The day of vaginal plug discovery was designated postconception day 0 (PC0). Gestational age was confirmed using external morphological features at necropsy (Theiler 1989). Randomly cycling, age-matched virgin females were used. All animals were housed in individually ventilated cages, on beta-chip bedding, and maintained under controlled conditions of temperature (20°–24°), humidity (40–60%), and lighting (12 hr light: 12 hr dark). Mice were fed standard chow diet and distilled water *ad libitum*. All animal husbandry protocols were in accordance with the guidelines of the American Association for the Accreditation of Laboratory Animal Care (AAALAC). All experimental protocols conducted in this study were approved by the Florida State University Animal Care and Use Committee.

### RNA-seq library preparation

The brain tissues were dissected rapidly, flash-frozen in liquid nitrogen, and maintained on dry ice until storage at –80°. Frozen brain tissue was homogenized in 1 ml TRIzol solution (Life Technologies 15596-018), using a motorized homogenizer. Samples were then centrifuged at 4° for 10 min at 4000 rpm to separate out debris, and the resulting supernatant was processed for total RNA extraction. Total RNA was treated with TURBO DNase (Ambion AM223) and purified with Zymo Research RNA Clean & Concentrator-25kit (Zymo Research R1018). The DNase treated and purified total RNA (5 μg for cerebellum, neocortex, and hippocampus, or 1 μg for hypothalamus) was used for subsequent mRNA isolation and RNA-seq library preparation. The External RNA Controls Consortium (ERCC) RNA Spike-in Controls (Ambion 4456740) were diluted 1:10 (for 5 μg total RNA sample) and 1:100 (for 1 μg total RNA sample) and added to total RNA. RNA-seq libraries were generated using the NEBNext Ultra RNA Library Prep Kit for Illumina (New England Biolabs Inc. E7530L), with 12 PCR cycles. The qualities of the libraries were examined using Agilent High Sensitivity DNA Bioanalyzer Chips (Agilent Technologies 5067-4626) and quantified by KAPA Library Quantification Kits for Illumina sequencing platforms (KAPA Biosystems KK4824).

### Illumina sequence read mapping and analyses

Initial quality checks on the Illumina sequencing data were done using FastQC (version 0.11.2, Babraham Bioinformatics). Raw sequence reads were preprocessed using Trimmomatic to remove Illumina adaptor sequence (Bolger *et al.* 2014), retaining only sequences that are 50 bases or longer. The ILLUMINACLIP section of the Trimmomatic command was set to two seed mismatches, thus allowing a maximum of two mismatches to be allowed while still allowing a full match to the adapter sequence, a palindromeClipThreshold of 30, and a simpleClipThreshold of 10.

The *Mus musculus* reference genome (release 75) was obtained from Ensembl (Flicek *et al.* 2014). The vast majority of the Illumina sequence data resulted in reads that are 100 bases in length after processing. The processed Illumina reads were mapped to the reference genome using the Tophat alignment tool (Trapnell *et al.* 2009), version 2.0.8, which uses Bowtie 2 as its underlying alignment algorithm. The parameters of Tophat were set to their default values, except the number of allowed mismatches and allowed edit distance (–read-mismatches and –read-edit-dist), which was set to six. The –GTF option was used to provide Tophat with a set of gene model annotations. easyRNASeq (obtained from Bioconductor version 3.0.2) was used to extract a count table from the Tophat files (Delhomme *et al.* 2012), containing raw count data for the genic feature of interest (gene), used in the count option and the geneModels option in the summarization parameter. The chromosome sizes option was set to auto to derive the chromosome sizes from the header of the BAM file generated by Tophat.

Cufflinks (version 2.1.1) was used to determine FPKM (Fragments Per Kilobase of Exon Per Million Fragments Mapped) values for each gene, based on the obtained full length and junction reads, utilizing the same GTF gene annotation file as before, and the –no-effective-length-correction flag set (Trapnell *et al.* 2012). The FPKM values were used to filter the final differentially expressed gene lists. Here, we require that for a gene to be considered in our statistical analyses, it needed to have a minimum FPKM value of 1, in all three replicates, in at least one treatment condition.

We used the edgeR statistical package (Bioconductor version 3.0.2) (Robinson *et al.* 2010). The trimmed means of M normalization method was performed on all samples. To determine if a gene is differentially expressed, we used the “tagwise” model of calculating dispersion. The p values were converted to q values by applying a false discovery rate (FDR) correction to account for multiple testing and false positives (Benjamini and Hochberg 1995). Genes that were determined to be significantly differentially expressed are provided in Supporting Information, Figure S1, Table S6, Table S7, Table S8, Table S9, Table S10, Table S11, Table S12, Table S13, Table S14, Table S15, Table S16, Table S17, Table S18, Table S19, and Table S20.

DESeq2 (obtained from Bioconductor version 3.0.2) was used to obtain PCA plots of the replicates (Love *et al.* 2014). The PCA plot generates two-dimensional plots of the first two components of the replicates for all the samples, allowing us to visualize the variance among the replicates. We note that we observe differences in variance among our biological replicates from the different time points (Figure S15). Thus, we do not have the same power to detect differences in all samples, due to this variance. Furthermore, we examined two PC time points and three PP time points, so we have different power to detect differences in the PP stages.

### Clustering approaches

Validation of our dissections was conducted by performing a literature survey (Sandberg *et al.* 2000) and identifying brain-region-specific genes in the Allen Brain Atlas (Lein *et al.* 2007). Visualization of our gene lists was done by generating heat maps using the hierarchical clustering module of GENE-E (http://www.broadinstitute.org/cancer/software/GENE-E/). The FPKM values that were obtained from Cufflinks were averaged, for each time point, for each gene for the hierarchical clusters. GENE-E was also used to find expression pattern similarities among the 49 genes that are common in the differentially expressed lists among all comparisons using K-means clustering. We found an initial value of nine for clustering centroids was optimal.

WebGestalt was used for gene ontology (GO) enrichment analysis and visualization of the ontology trees (Zhang *et al.* 2005). Genes determined to be differentially expressed in the pairwise comparisons examined were collated into gene lists that were classified as being induced or repressed. These gene lists were tested against the full mouse genome, using a hypergeometric test, to identify significantly enriched terms (p value <0.05 after a Benjamini-Hochberg correction). Ensembl gene IDs were provided to the WebGestalt software. Each GO analysis (biological processes, molecular functions, and cellular components), Phenotype ontology, and KEGG (Kyoto Encyclopedia of Genes and Genomes) pathway had to contain five or more genes to be considered enriched.

### Data availability

The RNA-seq data are available in the FASTQ format at the gene expression omnibus (GEO) with the accession number: GSE70732.

## Results and Discussion

### Experimental overview

**Figure 1:**
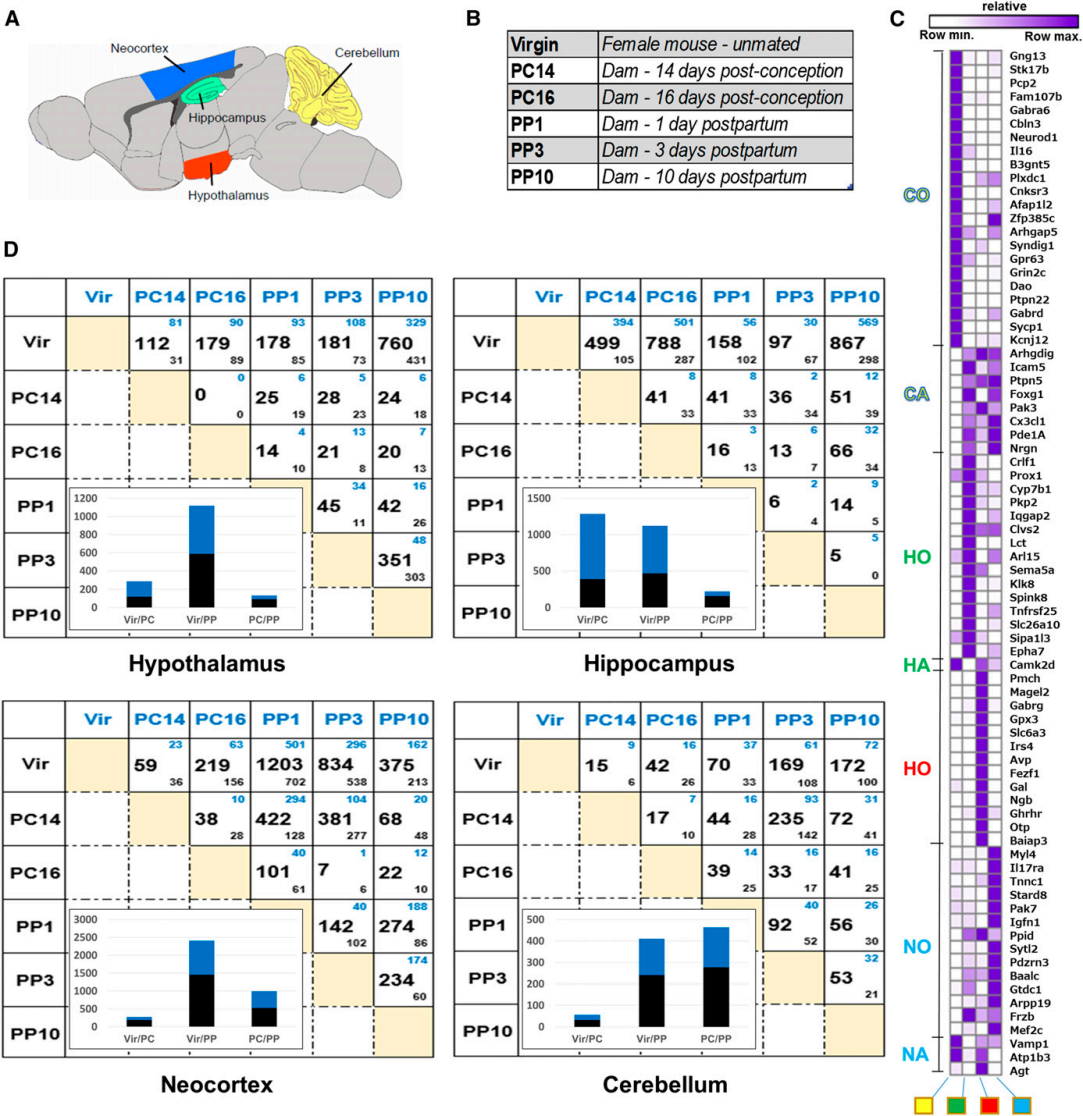
Transcriptome analyses of four regions of the mouse brain. (A) The four brain regions examined are: cerebellum (yellow); hippocampus (green); hypothalamus (red); and neocortex (blue). (B) Overview of the research design with six different stages examined: virgin females, two postconception (PC) stages, and three postpartum (PP) stages. (C) Hierarchical clustering of genes from literature survey and the Allen Brain Atlas data to check for region specificity of gene expression. The expected expression is indicated on the left of the cluster, as follows: cerebellum only (CO; yellow), cerebellum absent (CA; yellow), hippocampus only (HO; green), hippocampus absent (HA; green), hypothalamus only (HO; red), neocortex only (NO; blue), and neocortex absent (NA; blue). Columns are labeled at the bottom, from left to right: cerebellum (yellow square), hippocampus (green square), hypothalamus (red square), and neocortex (blue square). In the cluster, purple indicates row maximal expression, and white indicates row minimum expression. (D) Number of significant differentially expressed genes for each pairwise comparison (FDR corrected p value <0.05). Genes higher in the condition in blue (column labels) or black (row labels) are indicated by smaller blue and black numbers, respectively. Insets show the number of genes that are higher (blue) or lower (black) in virgin (Vir) and PC comparisons (Vir/PC), virgin and PP comparisons (Vir/PP), and PC and PP comparisons (PC/PP), respectively.

The goal of this study was to determine the gene expression changes in the female mouse brain that are a result of pregnancy, parturition, and the postpartum maternal experience using RNA-seq. We examined four brain regions in C57BL/6J (B6) female mice (hypothalamus, hippocampus, neocortex, and cerebellum; Figure 1A). Gene expression was compared in closely age-matched virgin females; females pregnant 14 and 16 days after observation of a seminal plug; and nursing dams with postnatal day 1, 3, and 10 pups (Figure 1B). Dams that are 14 and 16 days post observation of a seminal plug will be referred to as postconception days 14 and 16 (PC14 and PC16) (Figure 1B). Dams at postpartum days 1, 3, and 10 will be referred to as postpartum days 1, 3, and 10 (PP1, PP3, and PP10) (Figure 1B). The pregnancy (PC14 and PC16) and postpartum (PP1 and PP3) time points are equally spaced and also close to parturition, whereas the PP10 time point is when maternal behaviors are robustly displayed, thus analysis of these time points are likely to provide insight into the molecular-genetic basis of creating the potential for maternal behaviors, in the changing hormonal landscape surrounding parturition.

Samples of mRNA from three biological replicates were examined. For each replicate, mRNA was derived from a single mouse brain, with each mouse brain being used to collect all four brain regions. All sequencing libraries were confirmed to be of high-quality using FastQC (Andrews 2010). In addition, spike-in control mRNAs at a large range of concentrations were added at the total RNA step of library preparation (Jiang *et al.* 2011), and the results showed that all library preparation steps were of high quality (Table S1). Gene expression levels are reported as normalized values that take into account gene length and RNA-seq library size (Fragments Per Kilobase of Exon Per Million Fragments Mapped; FPKM) (Trapnell *et al.* 2012) (Table S2, Table S3, Table S4, and Table S5 for FPKM values for each brain region). Significant differential gene expression was determined using the statistical package edgeR (FDR-adjusted p value <0.05 was used in all analyses) (Robinson *et al.* 2010) (Table S6, Table S7, Table S8, Table S9, Table S10, Table S11, Table S12, Table S13, Table S14, Table S15, Table S16, Table S17, Table S18, Table S19, and Table S20).

To test the accuracy of the dissections, we compiled a list of genes that had been reported to have region-specific expression or absence of expression and examined their expression values in our data sets (Figure 1C, Table S1, and Table S21) (Lein *et al.* 2007; Sandberg *et al.* 2000). Genes previously implicated as having region-specific expression largely show the expected patterns here. For example, genes previously demonstrated as having only expression in the cerebellum (CO; Figure 1C) have high expression in the cerebellum (purple in CO rows; Figure 1C) and low or an absence of expression in other brain regions. Taken together with similar analyses of all four brain regions, we provide evidence that the dissections were performed accurately (Figure 1C).

### Global analyses of gene expression changes

Our results indicate that there are extensive changes in gene expression in all regions of the brain examined (Figure 1D). All females were closely age-matched, kept in the same environmental conditions, and were the same strain. Thus, we expect that a majority of gene expression differences observed here between virgin, pregnant, and postpartum females were due to the effects of pregnancy, parturition, and postpartum maternal experience. We refer to genes with higher expression in pregnant or nursing dams compared to virgins as induced (blue small number in top of boxes, Figure 1D), and those with lower expression as repressed (black small number in bottom of boxes, Figure 1D). In postconception (PC) *vs.* postpartum (PP) comparisons, genes with higher and lower expression in the PP stage are considered induced and repressed, respectively.

Examination of each pairwise comparison in our data sets (Figure 1D), as females progress from pregnancy to the postpartum period, shows that a large number of genes have expression differences, with each brain region examined having many differentially expressed genes: hypothalamus (1,043 genes; Table S24), hippocampus (1,243 genes; Table S23), neocortex (1,940 genes; Table S25), and cerebellum (606 genes; Table S22). Thus, the neocortex shows the most expression change and the cerebellum is the most stable. The large number of induced and repressed genes suggests that the transition to motherhood involves repression of some active pathways and induction of new pathways.

For the pairwise comparisons with virgin animals, there were generally more genes with differences in expression when compared to the PP stages than the PC stages (Figure 1D, histogram insets in lower left part of each box). One exception is the gene expression differences in the hippocampus, where both the virgin *vs.* PC and the virgin *vs.* PP comparisons show a large number of genes with expression differences. This suggests that the hippocampus is undergoing as many gene expression changes during pregnancy as during PP stages, whereas the cerebellum, hypothalamus, and neocortex have the most substantive changes compared to virgins during PP stages. It is clear from previous studies that there are cognitive changes throughout pregnancy that are mediated by the hippocampus, with improved cognition early in pregnancy, but during late pregnancy and early postpartum stages there was impaired spatial cognition (Galea *et al.* 2014); the gene expression data here may provide insight into those differences.

Interestingly, when we compare expression differences between PC and PP stages in each brain region, we see the largest number of differentially expressed genes in the cerebellum, whereas the other brain regions have the largest differences in the virgin comparisons *vs.* PC or PP stages. This suggests that, in the hippocampus, hypothalamus, and neocortex, many of the changes in gene expression arise during pregnancy and persist during PP stages, whereas in the cerebellum the genes that change expression during PC stages are different than those for the PP stages.

**Figure 2:**
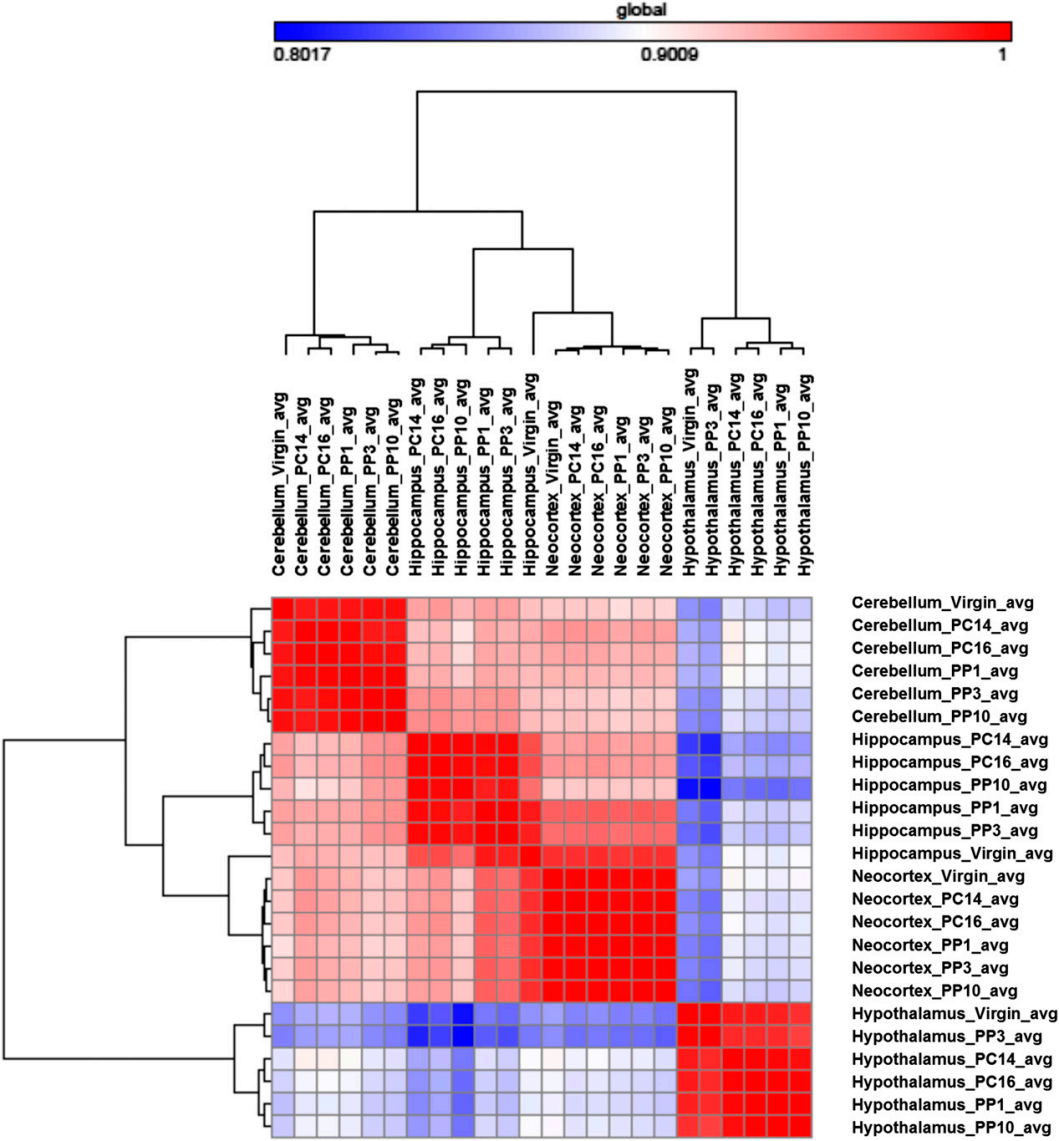
Heat map of sample distance and similarity matrix. The set of 3737 genes that showed differential expression in at least one pairwise comparison was used for this analysis. For each gene, the averaged FPKM (Fragments Per Kilobase of Exon Per Million Fragments Mapped) values were used to generate the similarity of expression plot between time points and across brain regions. The hierarchical cluster and the similarity matrix were generated by GENE-E, using the “one minus Pearson correlation coefficient” metric, with the average linkage method (http://www.broadinstitute.org/cancer/software/GENE-E/).

To determine which brain regions are most similar with respect to genes with significant expression changes, we made a correlation cluster using the data from the 3737 genes that had an expression change in any pairwise comparison (Figure 2). As expected, the data from within each brain region show the highest correlation across time points. Across brain regions, the neocortex and hippocampus show the highest correlation in gene expression levels, whereas the data from the hypothalamus is the least correlated with the other brain regions. These results fit well with the fact that both the hippocampus and neocortex have roles in memory, learning, and cognition, and with the unique role of the hypothalamus in hormone production and regulation of progression through reproductive stages.

### Similarities and differences in gene expression changes across the four brain regions

**Figure 3:**
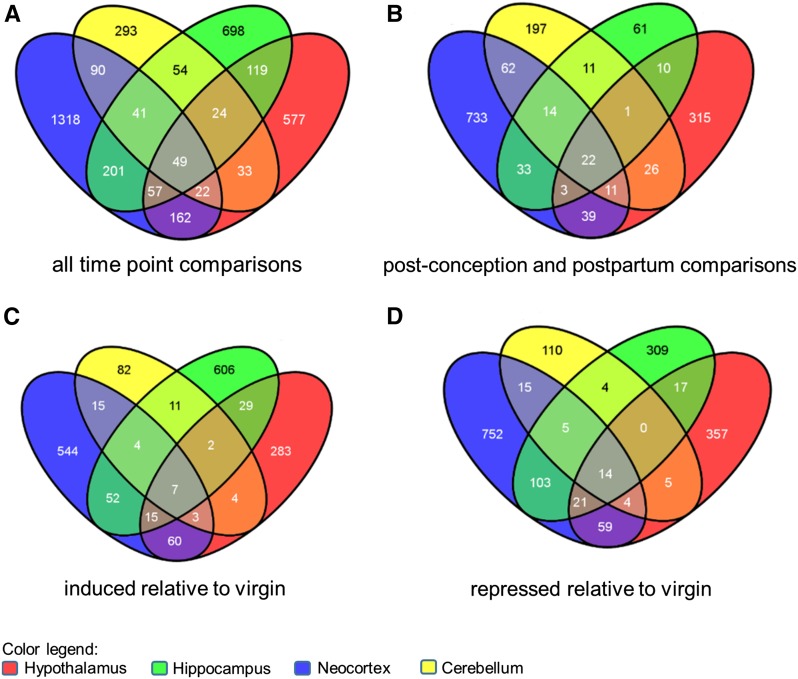
Brain regions have distinct and overlapping sets of differentially expressed genes. Venn diagrams illustrating the overlap of differentially expressed genes across the cerebellum (yellow), hippocampus (green), hypothalamus (red), and neocortex (blue) data sets. The diagrams were made using the online tool Venny (http://bioinfogp.cnb.csic.es/tools/venny/index.html). (A) A gene is included in this analysis if it was differentially expressed in any pairwise comparison. (B) A gene is included in this analysis if it was differentially expressed between or within any PC and PP time points. (C) A gene is included in this analysis if it was induced relative to virgin gene expression in a PC or PP time point. (D) A gene is included in this analysis if it was repressed relative to virgin gene expression in a PC or PP time point.

To determine if the four brain regions examined have unique gene expression differences, we examined the overlap of genes with differential expression across the four brain regions (Figure 3). We first examined all the genes that had differential expression in any time point comparison (Figure 3A), as described above (Figure 1D). We find that only 49 genes have differential expression in all four brain regions (Table S26), whereas 293 (cerebellum), 577 (hypothalamus), 698 (hippocampus), and 1318 (neocortex) genes are differentially expressed in only one brain region.

To understand the gene expression differences during pregnancy and the postpartum period, we also identified the genes with differences between any PP and PC time point, and determined the overlap (Figure 3B). Here we find that 22 of the 49 genes are differentially expressed in all brain regions (Table S26). Fewer genes are differentially expressed in each brain region than when the virgin comparisons were also included (Figure 3A), though generally there are fewer genes with differential expression in these comparisons (see insets in Figure 1D).

Finally, if we limit our analyses to genes that are either induced (Figure 3C) or repressed (Figure 3D) relative to virgins, we find only seven and 14 genes, respectively, that are differentially expressed in all four brain regions (Table S26). The seven genes that are induced includes four hemoglobin genes (*Hbb-bs*, *Hbb-bt*, *Hba-a1*, *Hba-a2*), and *aminolevulinic acid synthase 2* (*Alas2*)—the first enzyme in heme biosynthesis (Ponka 1999). This suggest that one commonality across all brain regions is a need for more hemoglobin, and perhaps an increase in blood flow or angiogenesis during PC and PP stages, relative to virgins, though we interpret this result with caution given that the brains were not perfused before tissue collection.

The 14 genes that are repressed include eight that are annotated with the GO term positive regulation of transcription from RNA polymerase II promoter. This includes members of the “early-response genes” including activating protein complex 1 (AP-1); encoded by *FBJ osteosarcoma oncogene* (*Fos*) and *JunB*, and two nuclear receptor superfamily members (*Nr4a1* and *Nr4a3*) (reviewed in West and Greenberg 2011); previous studies have shown that *fosB* mutant mice have a defect in maternal nurturing behaviors (Brown *et al.* 1996). Additionally, we found that two transcription factors that underlie aspects of circadian biology, *Per1* and *D site albumin promoter binding protein* (*DBP*), are repressed. Taken together, each brain region has a unique gene expression signature during the peripartum period, but also several overlapping genes.

### Similarities and differences in gene expression changes within each of the four brain regions

**Figure 4:**
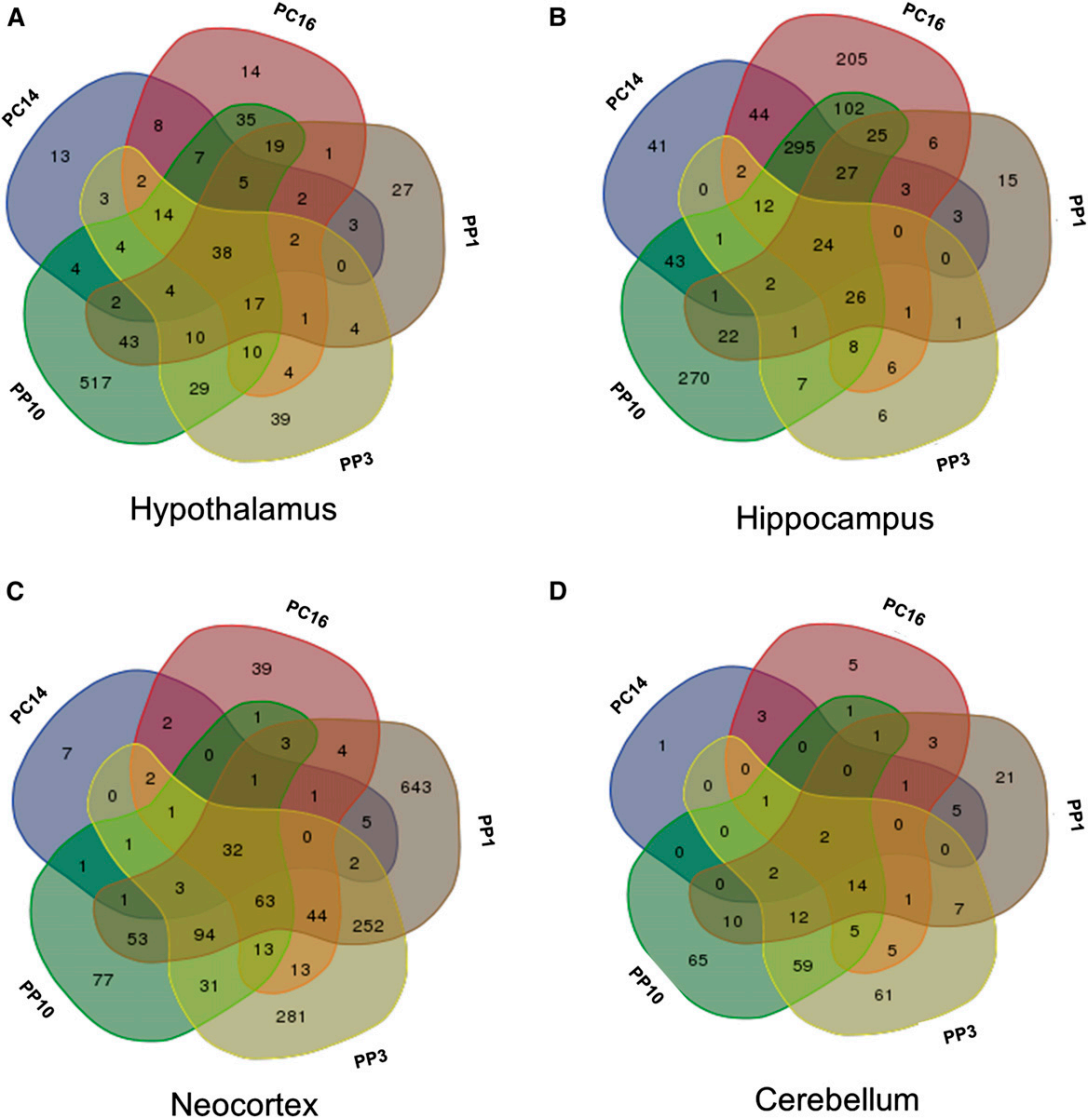
Time points have distinct and overlapping sets of differentially expressed genes. Overlap of the differentially expressed genes in each virgin pairwise comparison to PC and PP stages in the (A) hypothalamus, (B) hippocampus, (C) neocortex, and (D) cerebellum. The Venn diagram was made using an online tool (http://bioinformatics.psb.ugent.be/webtools/Venn/).

To determine if each time point examined has a unique gene expression response within each brain region, we determined for each brain region the overlap of differentially expressed genes among the five time points, each compared to virgins. For each brain region examined, each time point had a unique molecular signature (Figure 4). For the hypothalamus and cerebellum, later time points had progressively more unique differentially expressed genes than earlier time points (Figure 4, A and D). The hippocampus has the most unique gene expression differences at the PC16 and PP10 time points, whereas in the neocortex the most unique differences are observed at PP1 and PP3. Interestingly, time points that are closer together do not necessarily have more genes with differential expression in common.

We also examined similarities and differences in gene expression compared to virgins, across the four brain regions at each time point. We found that each brain region has a unique molecular response at each time point comparison examined, with the hippocampus and neocortex having the most genes with expression differences in common (Figure S1).

### Patterns of expression for genes with differential expression in all brain regions

**Figure 5:**
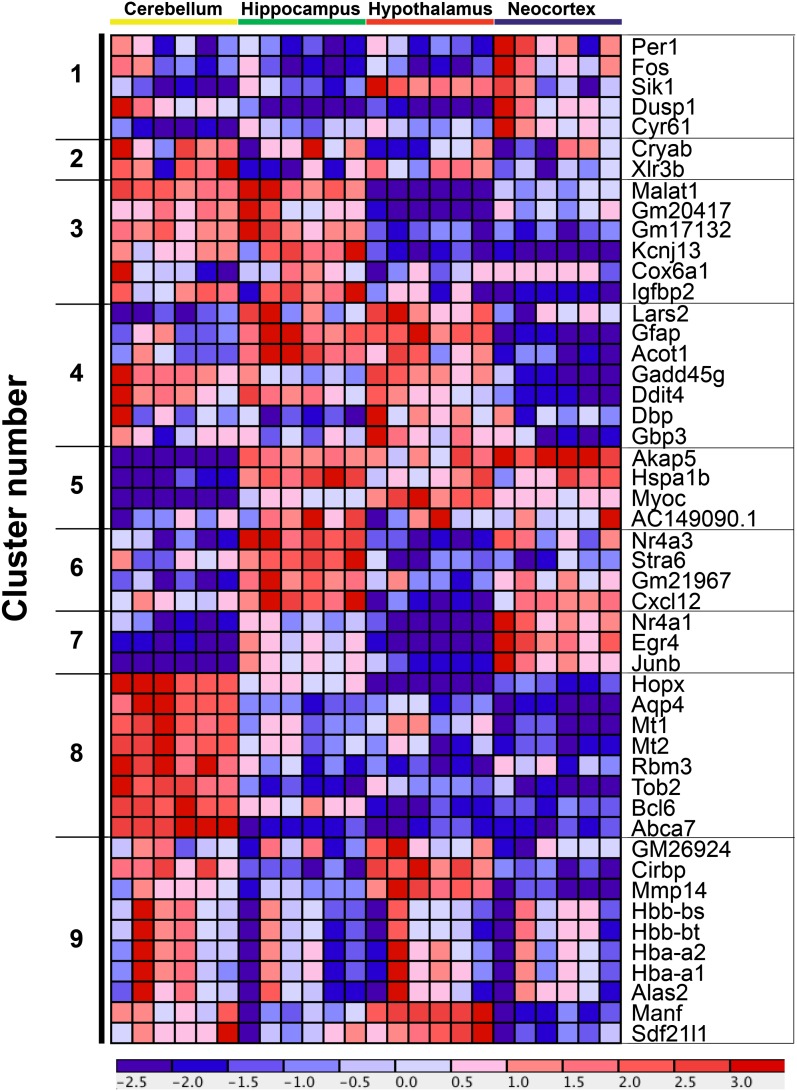
Expression pattern for the 49 genes that are differentially expressed in all four brain regions. Expression profile of the 49 genes found to have differential expression in all four brain regions (see Figure 3A). K-means clustering was performed by rows (the rows are the 49 genes indicated on the right of clustergram), resulting in 9 nodes of the cluster (indicated on the left of clustergram). The algorithm used row normalization and the Euclidean distance metric. The output is visualized with a cluster with red indicating high expression and blue indicating low expression.

We performed K-means clustering and visualization to determine if the 49 genes with differential expression in all four brain regions had similarities in expression (Figure 3A; highlighted in gray), with nine nodes as the optimum number. While the genes within a node show the same trends of mRNA fluctuation, for a given gene, each brain region is unique with respect to abundance. For example, in cluster nine, the hemoglobin genes, *Hbb* and *Hba*, have the same trends in terms of peak and trough expression, but the cerebellum generally has higher expression than the other brain regions across time points (Figure 5, Figure S2, and Figure S3). Similarly, genes in cluster 8 have higher expression in the cerebellum, but genes in clusters five, six, and seven tend to have higher expression in the hippocampus and neocortex. Thus, while the overall trends or patterns are similar across brain region, for each gene within a cluster, the overall abundance within each brain region is different.

Given that these 49 genes are differentially expressed in all brain regions, they represent a high confidence set, in terms of changing expression during PC and PP stages. We determined if homologs of the genes in this set have been previously implicated in underlying mental health disorders, especially depression, and provide annotation on what is known (Table S27). Overall, 21/49 have previously been implicated in underlying depression or other mental health disorders.

### Phenotype pathway, KEGG pathway, and GO enrichments

**Figure 6:**
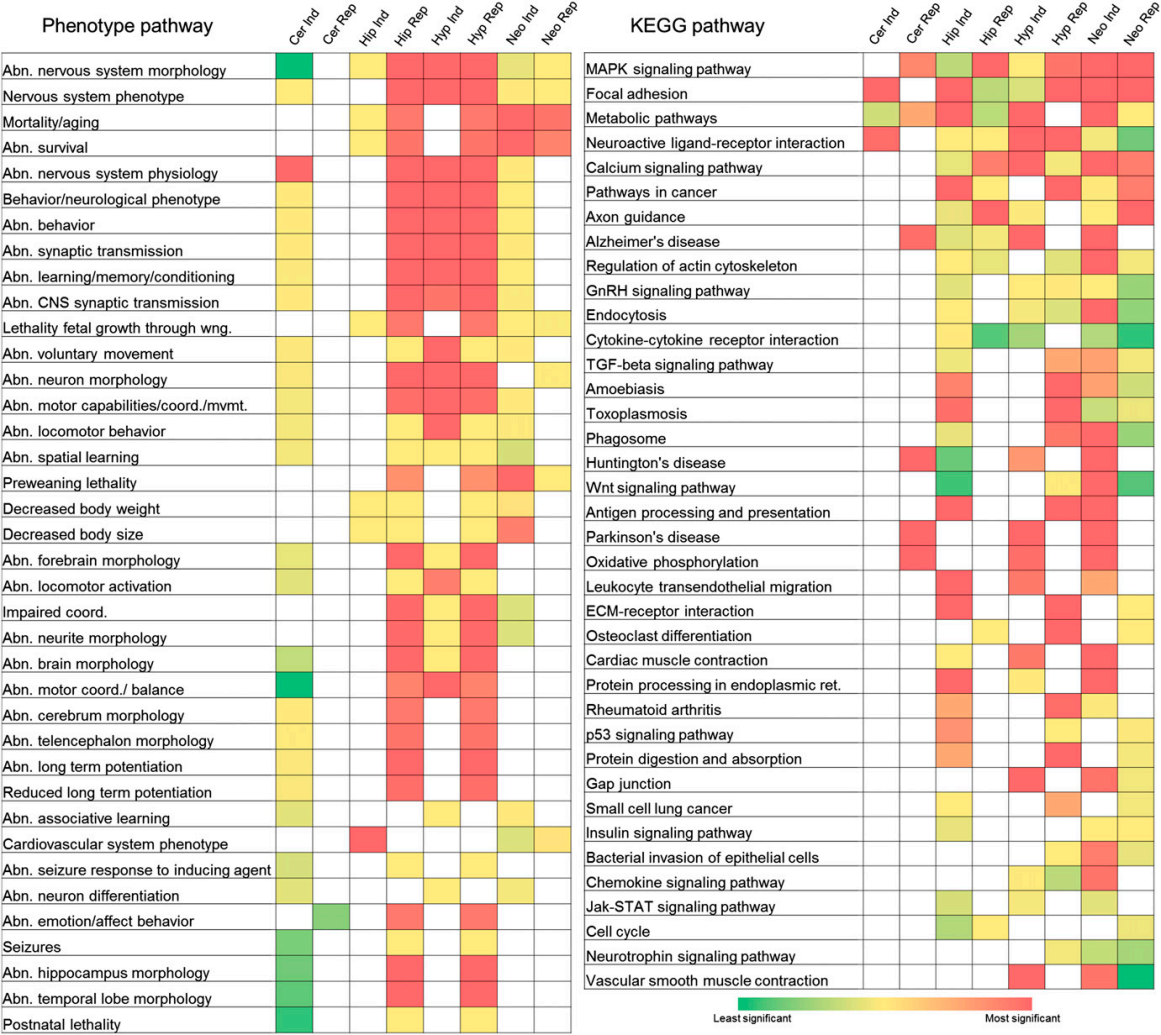
Phenotype ontology and KEGG ontology enrichments. The differentially expressed genes were separated into genes that were either induced (Ind) or repressed (Rep) with respect to virgins and analyzed for significantly enriched phenotype pathways and KEGG (Kyoto Encyclopedia of Genes and Genomes) pathways. The brain region abbreviations are: cerebellum (Cer), hippocampus (Hip), hypothalamus (Hyp), and neocortex (Neo).

We next determined for each brain region if the large numbers of genes that are induced or repressed at any time point relative to virgins (see Figure 1), are enriched with genes with phenotype ontologies, genes in KEGG pathways (Kanehisa and Goto 2000), or with GO terms, as assessed using the online portal WebGestalt (Zhang *et al.* 2005). First we looked for phenotype ontology enrichments that are prevalent across all brain regions (Figure 6); here we only show the phenotype ontologies that are significant in at least three analyses (Benjamini-Hochberg FDR corrected p value of <0.05), but the full data set is provided, with many additional interesting pathways to further study (Table S28).

We found several expected enriched phenotype ontologies, such as abnormal nervous system morphology and nervous system phenotype, as having the largest number of brain regions showing these as significant. The genes with repressed expression in the hippocampus and hypothalamus have the most shared, highly significant phenotype ontology enrichments (see filled red boxes in Figure 6), whereas genes repressed in the cerebellum were not enriched for phenotype ontologies common to other brain regions (Figure 6). Genes that are induced or repressed in the neocortex are highly enriched for genes that affect aging and survival, whereas only repressed genes in the hippocampus and hypothalamus are highly enriched for these phenotype ontologies. Among the phenotype ontologies common to genes differentially expressed in all four brain regions, genes induced and repressed in the hypothalamus and repressed in the hippocampus had the highest number of shared highly significant enrichments for these phenotype pathways. The observation that many brain regions have genes with differential expression are enriched for behavior, neuronal physiology, brain and neuronal morphology, and learning and memory ontologies, suggests that the developmental transition of the dam during pregnancy and parturition involves many complex processes acting across the brain.

Next we examined KEGG pathway enrichments that are prevalent across all brain regions (Figure 6; Table S28). The map kinase pathway is significantly enriched for genes identified in all four brain regions, for both induced and repressed genes, with the exception of genes induced in the cerebellum. This suggests that signal transduction is one of the primary mechanisms to mediate changes in all brain regions at the stages examined. Three other KEGG pathways that are highly enriched across nearly all brain regions, for both induced and repressed genes are: (1) focal adhesion, (2) metabolic pathways, and (3) neuroactive ligand-receptor interaction. The observation that focal adhesion and neuroactive ligand-receptor interaction are both enriched pathways in many brain regions, suggests that signaling either through the extracellular matrix or transmembrane receptors is important during pregnancy, parturition, and the postpartum period. Consistent with the notion that this transition is both a developmental and physiological transition, we observe additional axon guidance, developmental and physiological pathways as enriched pathways in many brain region data sets.

We also examined GO enrichments (biological, molecular, cellular) that are prevalent across all brain regions (Table S29). As expected, broad GO terms with many subset GO terms are highly enriched. Since those terms do not provide much insight, we focus on terms that are more specific, but are also among the most significant. Genes with induced and repressed differential expression in all brain regions are enriched for nerve impulse (biological process), ion transmembrane transporter activity (molecular function), and neuron projection (cellular process) (Table S29).

To determine if the molecular changes that underlie pregnancy are different than those that underlie parturition and the postpartum period, we partitioned the differentially expressed genes to those that are either different between virgins *vs.* PC stages; or virgins *vs.* PP stages; or PC *vs.* PP stages. In these analyses, genes that changed expression in the virgin and PP stages in the hippocampus, hypothalamus and neocortex share the most enriched phenotype pathway terms. The KEGG pathways that have the most shared highly significant enrichments in these comparisons also include the MAPK signaling pathway, metabolic pathways, neuroactive ligand-receptor interactions, and focal adhesions (Table S30)

### Brain region-specific patterns of gene expression changes

To understand the biologically important and robust changes in gene expression that occur within each brain region, we examined hierarchal clusters of genes that showed both significant and substantial differences (>two-fold difference in at least one comparison) (Figure S4, Figure S5, Figure S6, and Figure S7). We identified different numbers of genes in each brain region that meet these criteria: cerebellum (92 genes), hippocampus (196 genes), hypothalamus (180 genes), and neocortex (89 genes) (Table S31, Table S32, Table S33, and Table S34). The hierarchical cluster visualization was annotated with enriched GO terms, Disease and Pathway categories, as well as whether the gene shows region-specific changes in expression, to gain more insight.

The majority of genes within each cluster were unique to that brain region cluster (last column of each cluster annotation; Figure S4, Figure S5, Figure S6, and Figure S7). Further, the GO terms were distributed throughout the clusters, demonstrating that genes that are coexpressed are from diverse functional groups. All the clusters had subclusters containing the four hemoglobin genes (*Hbb-bs*, *Hbb-bt*, *Hba-a1*, *Hba-a2*) and *Alas2*, with the same overall patterns of expression. Additionally, all four clusters have subclusters that contain genes encoding transcription factors, including “early-response genes”, that start out with high expression in virgins and then show lower expression during PC and PP stages. The transcription factors include *cysteine rich protein 61* (*Cyr61*), *Dbp*, *Fos*, *fos-like antigen 2 Fosl2*, *Kruppel-like factor 2* (*Klf2*), *neuronal PAS domain protein 4* (*Npas4*), and *nuclear receptor subfamily 4*, *group A members 1*, *2*, and *3* (*Nr4a1*, *Nr4a2*, and *Nr4a3*). Five of these transcription factors have been annotated with the GO category response to steroid (*Fosl2*, *Npas4*, *Nr4a1*, *Nr4a2*, and *Nr4a3*).

For each brain region we also examined the genes with maintained induced or repressed significant expression differences relative to virgins (FDR <0.05 in all virgin comparisons and either induced in all or repressed in all comparisons). The hypothalamus has the largest number of genes with sustained induced expression (Figure S9, Figure S10, and Figure S11) and the neocortex has the largest number of genes with sustained repressed expression (Figure S12, Figure S13, and Figure S14), with several “early response genes” showing maintained repressed expression in the hypothalamus, hippocampus, and neocortex. The cerebellum only had one gene that maintained repressed expression (*Insulin-like growth factor binding protein*; *Igfbp4*) and none with maintained induced expression.

Additionally, we determined if each brain region had a different repertoire of genes with significant expression differences between the postconception stages PC14 and PC16, the two pregnancy time points in this study. We find that the data from the hypothalamus has no genes with significant expression differences between PC14 and PC16, despite seeing substantive changes in other time point comparisons, suggesting that many of the expression differences at PC14 are maintained at PC16, or that we lack sufficient power to detect the changes. For the other three brain regions, there were more genes with significantly higher expression at PC14 compared to PC16, suggesting that repression of gene expression is an important mechanism for the transition to the late pregnancy time points (Table S6 and Table S36).

### Common and distinct molecular function GO enrichments

**Figure 7:**
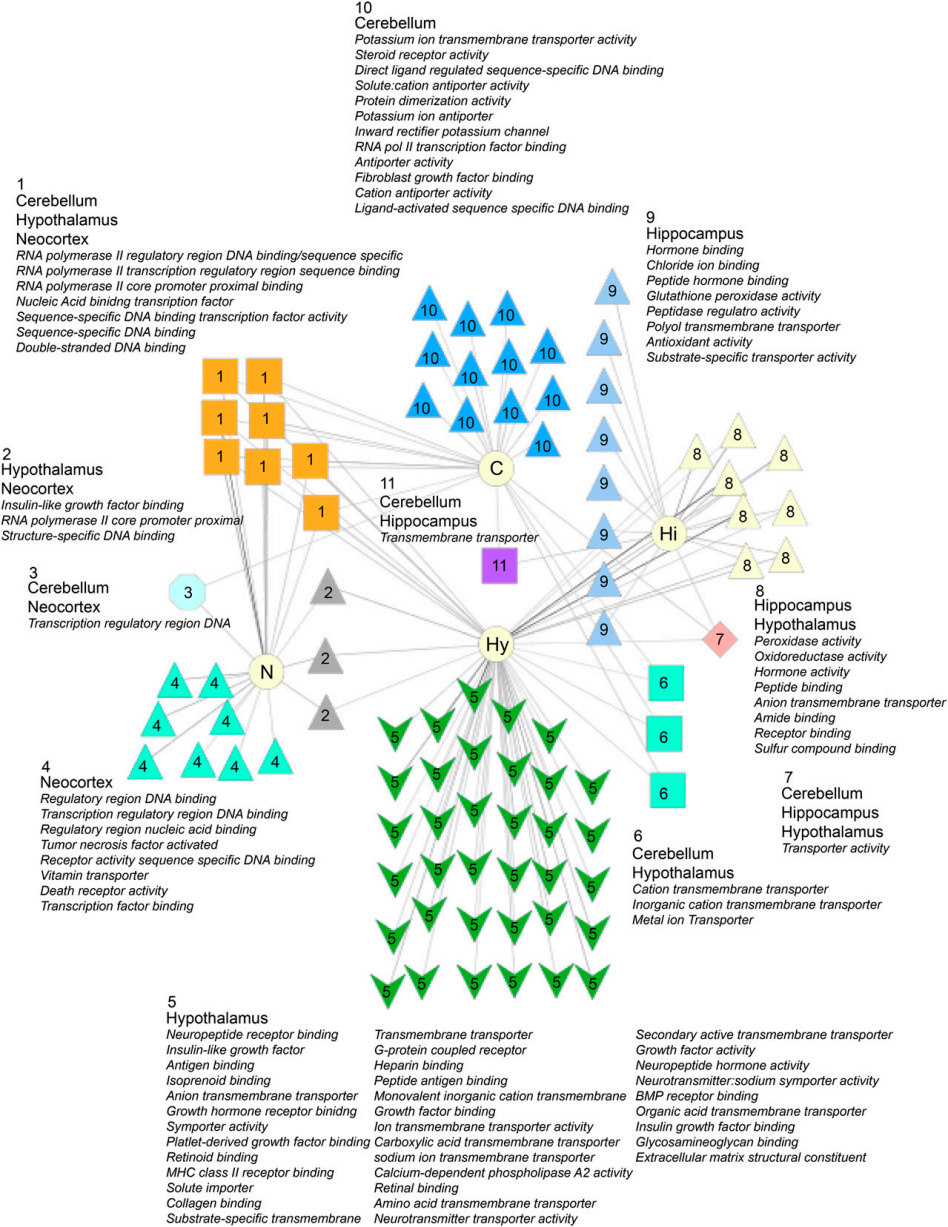
Molecular ontology enrichments in the four brain regions. Genes with significant and substantial gene expression differences were identified for each brain region examined (FDR <0.05; >two-fold difference in at least one comparison). Toppcluster was used to find significant molecular gene ontology (GO) enrichments for each brain region and their overlap. For an ontology to be considered, it needed to include at least five genes at an FDR cut-off of <0.05.

We used ToppCluster, a clustering tool that finds overlapping ontology enrichments in several gene lists (Kaimal *et al.* 2010), to identify shared and distinct GO molecular enrichments in the lists of genes with significant and substantial differences (FDR <0.05 and >two-fold difference in expression in at least one comparison; Figure 7). The hypothalamus has the most unique molecular ontologies (green arrowheads, Figure 7). These unique ontologies include insulin-like growth factor, G-protein coupled receptor, neuropeptide receptor binding, neurotransmitter transporter activity, bone morphogenetic protein binding, and many others. The cerebellum, hypothalamus, and neocortex share several molecular ontology enrichments for transcription factor molecular terms (orange boxes, Figure 7). We find that the neocortex uniquely has tumor necrosis factor and death receptor activity as GO terms, whereas the hippocampus has hormone binding as a unique term. Taken together, these results further demonstrate that while there are many shared molecular changes in the four brain regions, each region also has a unique molecular signature.

### Genes previously shown to have a role in maternal behaviors

We performed a literature search to find genes previously implicated in the developmental transition to motherhood, and examined the expression data (Table S35; references therein). For this we report all FPKM values obtained, but, for all of our statistical analyses above, a gene was considered only if it had FPKM values >1 for all three samples in at least one treatment group of the comparison. A hierarchal cluster of the data shows the interesting expression patterns of these genes (Figure S8).

*Oxytocin* (*Oxt*) is one of the most critical genes known for maternal behaviors, playing key roles in parturition and lactation (Pedersen *et al.* 1994; Rich *et al.* 2014; Russell *et al.* 2003). We find that *Oxt* shows significant differences in expression in the hypothalamus, but not in other tissues examined. In the hypothalamus, *Oxt* has high expression in virgins (648 FPKM), trough expression in PC14 (346 FPKM), and then rising, progressive elevated expression in PP stages, but not substantially higher than observed in virgins (652 FPKM at PP10). Furthermore, the *oxytocin receptor* (*Oxtr*) does not significantly change expression in any of the brain regions examined, nor is expression high in any tissue. The average expression is highest in the hypothalamus (2.89 FPKM), lowest in the cerebellum (0.19 FPKM), and intermediate in the hippocampus (1.98 FPKM), and Neocortex (1.04 FPKM).

On the other hand, *prolactin* (*Prl*), important for postpartum mood and behavior (Bridges *et al.* 1990; Larsen and Grattan 2012), has significant changes in expression in all brain regions examined, with relative peak expression at PP1 and PP3, in all brain regions examined. The hypothalamus had the largest difference in peak and trough expression, with virgins having the lowest expression (0.11 FPKM), and PP3 having the highest expression (74.24 FPKM). This expression pattern was similar to *growth hormone* (*Gh*), which also has been shown to have fluctuating levels from several different sources depending on reproductive state (Hull and Harvey 2002).

Interestingly, the prolactin receptor (*Prlr*) shows significant changes in expression in three brain regions, but not the neocortex. The cerebellum and hippocampus start out with trough expression of *Prlr* in virgins (0.94 FPKM and 1.03 FPKM), and peak expression at PP10 (4.92 FPKM and 11.19 FPKM), with a five-fold and 11-fold difference in expression, respectively. In the cerebellum and hippocampus, elevated expression relative to virgins is observed at all PC and PP stages. The hypothalamus shows a different pattern, with significant differences in expression only between PP1 (peak expression, 2.6 FPKM) and PP10 (trough expression, 1.91 FPKM). The gene *prolactin releasing hormone* (*Prlh*) is not detectably expressed at any time point examined in any tissue, except the hypothalamus, whereas the *prolactin releasing hormone receptor* (*Prlhr*) is detectable at low FPKM values in all tissues, but has highest expression in the hypothalamus.

### Genes implicated in depression

Given the relatively high incidence of postpartum depression (30–75% have postpartum blues), and other mental health disorders in women after pregnancy (reviewed in Andersson *et al.* 2006; Josefsson *et al.* 2001; Philipps and O’Hara 1991; Stein *et al.* 1981), we examined the full data set to determine if there is any overlap of genes with expression changes during pregnancy and the postpartum period, with genes implicated in human postpartum depression or depressive disorders. We used the HuGE Navigator to find human genes that are associated with these terms (Yu *et al.* 2010). For the disease term *postpartum depression*, there are 17 human genes in the database, for which we found 16 mouse homologs. Each brain region had a set of these genes with differential expression (Table S37): cerebellum (three genes), hippocampus (two genes), hypothalamus (six genes), and neocortex (four genes). For the disease term *depressive disorder*, there were 502 human genes, for which we found 475 mouse homologs. Each brain region also had a set of these genes with differential expression (Table S37): cerebellum (29 genes), hippocampus (51 genes), hypothalamus (47 genes), and neocortex (67 genes). There were three genes that were differentially expressed in all four brain regions: *Per1*, *Dbp*, *heat shock protein 1B* (*Hspa1b*).

We note, however, that we do not see a significant enrichment of depression-related genes in our mouse phenotype ontology analyses of all genes with differential expression or genes with region-specific differential expression in this study, using the ToppCluster portal, but anxiety-related, parental behavior, decreased dopamine, and abnormal emotion/affect behavior phenotype gene categories were enriched. Thus, in the mouse, it will be interesting to determine if postpartum mental-health related phenotypes manifest, in part, as anxiety phenotypes. The expression data set presented here may also reveal new candidate genes, pathways, and networks to investigate their roles in human postpartum depression, which is poorly understood and may be different than other categories of depression, with differences in the underlying molecular and neuroanatomical etiology. In addition, the large numbers of differentially expressed genes in hippocampus both prepartum and postpartum suggest that there is significant reorganization in the gene expression profile of this structure, which has been implicated as having a key role in depression (reviewed in Pawluski *et al.* 2015).

### Conclusions

It is clear that the brain undergoes extensive pregnancy and postpartum changes in gene expression in the four regions that we examined. We find that each brain region had a unique molecular response, with several hundred genes changing expression; however, we also found a core set of 49 genes that change expression in all four brain regions. The hippocampus and hypothalamus had the largest number of genes that showed significant and robust changes, whereas the cerebellum had the fewest. In all four brain regions there is a set of some of the “early-response gene” transcription factors implicated in diverse processes that start out with high expression in virgin females, then have reduced expression through pregnancy and the postpartum period.

The study presented here is only a subset of the types of information that these data sets can yield, with additional future analyses to provide further insights. In the future, it will also be interesting to examine how extensive these gene expression changes are within each brain region. An examination of the *in situ* expression of these genes can contribute to the identification of neural circuits that underlie maternal behaviors and help to determine if there are dedicated circuits for maternal behavior or if the potential for this behavior is distributed throughout the brain. This study also provides a comprehensive transcriptional atlas of the maternal hypothalamus, hippocampus, neocortex, and cerebellum during late pregnancy and the early postpartum period that can be leveraged for future studies to examine maternal behaviors, and also to understand how the normal perturbation of gene pathways during pregnancy and postpartum stages may lead to human postpartum depression. Future studies examining additional time points both early in pregnancy and later postpartum time points will be important additions to this data set to understand the dynamic changes underlying these developmental transitions.
